# Dual-Functionalized Mesoporous Silica Nanoparticles for Celecoxib Delivery: Amine Grafting and Imidazolyl PEI Gatekeepers for Enhanced Loading and Controlled Release with Reduced Toxicity

**DOI:** 10.3390/molecules29153546

**Published:** 2024-07-27

**Authors:** Diky Mudhakir, Ebrahim Sadaqa, Zuliar Permana, Jihan Eldia Mumtazah, Normalita Faraz Zefrina, Jovinka Natalie Xeliem, Latifa Fawzia Hanum, Neng Fisheri Kurniati

**Affiliations:** 1 Department of Pharmaceutics, School of Pharmacy, Institut Teknologi Bandung (ITB), Bandung 40132, Indonesia; 30722701@mahasiswa.itb.ac.id (E.S.); 30720315@mahasiswa.itb.ac.id (Z.P.); 10719008@mahasiswa.itb.ac.id (J.E.M.); 20721037@mahasiswa.itb.ac.id (N.F.Z.); 10720011@mahasiswa.itb.ac.id (J.N.X.); 10720105@mahasiswa.itb.ac.id (L.F.H.); 2 Department of Clinical and Community Pharmacy, School of Pharmacy, Institut Teknologi Bandung (ITB), Bandung 40132, Indonesia; nfkurniati@itb.ac.id

**Keywords:** mesoporous silica nanoparticles, anti-inflammatory agent, imidazolyl-PEI, loading drug, cytotoxicity

## Abstract

The development of targeted drug delivery systems has been a pivotal area in nanomedicine, addressing challenges like low drug loading capacity, uncontrolled release, and systemic toxicity. This study aims to develop and evaluate dual-functionalized mesoporous silica nanoparticles (MSN) for targeted delivery of celecoxib, enhancing drug loading, achieving controlled release, and reducing systemic toxicity through amine grafting and imidazolyl polyethyleneimine (PEI) gatekeepers. MSN were synthesized using the sol–gel method and functionalized with (3-aminopropyl) triethoxysilane (APTES) to create amine-grafted MSN (MSN-NH_2_). Celecoxib was loaded into MSN-NH_2_, followed by conjugation of imidazole-functionalized PEI (IP) gatekeepers synthesized via carbodiimide coupling. Characterization was conducted using Fourier-transform infrared spectroscopy (FTIR) and proton nuclear magnetic resonance (^1^H-NMR). Drug loading capacity, entrapment efficiency, and in vitro drug release at pH 5.5 and 7.4 were evaluated. Cytotoxicity was assessed using the MTT assay on RAW 264.7 macrophages. The synthesized IP was confirmed by FTIR and ^1^H-NMR. Amine-grafted MSN demonstrated a celecoxib loading capacity of 12.91 ± 2.02%, 2.1 times higher than non-functionalized MSN. In vitro release studies showed pH-responsive behavior with significantly higher celecoxib release from MSN-NH_2_-celecoxib-IP at pH 5.5 compared to pH 7.4, achieving a 33% increase in release rate within 2 h. Cytotoxicity tests indicated significantly higher cell viability for IP-treated cells compared to PEI-treated cells, confirming reduced toxicity. The dual-functionalization of MSN with amine grafting and imidazolyl PEI gatekeepers enhances celecoxib loading and provides controlled pH-responsive drug release while reducing systemic toxicity. These findings highlight the potential of this advanced drug delivery system for targeted anti-inflammatory and anticancer therapies.

## 1. Introduction

Nonsteroidal anti-inflammatory drugs (NSAIDs) are widely used for managing inflammatory conditions due to their analgesic, antipyretic, and anti-inflammatory properties [1]. Among these, celecoxib, a selective COX-2 inhibitor, is commonly prescribed for osteoarthritis and rheumatoid arthritis and has demonstrated anticancer efficacy by selectively inducing apoptosis in malignant cells [2,3]. However, the clinical use of celecoxib is limited by challenges such as poor solubility, low bioavailability, and systemic toxicity, necessitating the development of advanced drug delivery systems [4,5,6]. MSN have emerged as promising carriers for hydrophobic drugs, including celecoxib, due to their high drug loading capacities and ability to precisely control release kinetics [7,8]. The unique mesoporous structure of MSN allows for the encapsulation of hydrophobic drugs within the silica matrix, while their highly tunable surface chemistry enables the conjugation of polymers and ligands to improve loading and tailor release profiles [9,10,11].

Several surface engineering strategies have been explored to enhance the incorporation of celecoxib into MSN. These include amine grafting to increase pore volumes and functionalizing exteriors with cationic polymers or stimuli-responsive moieties [12,13,14]. Grafting amino silanes such as APTES has been shown to significantly increase drug loading by expanding pore diameters in MSN [15,16]. In a study conducted by She et al., hollow mesoporous silica nanoparticles (HMSNs) were functionalized with various chemical groups, including amine (-NH_2_), carboxyl (-COOH), cyano (-CN), and methyl (-CH_3_) groups, to investigate their effect on the loading capacity of the anticancer drug 5-fluorouracil (5-FU). Interestingly, the presence of amine groups on the surface of nanoparticles resulted in the highest loading capacity of 28.89%, compared to other functional groups. However, while amine functionalization can increase loading capacity, it may not provide sufficient control over drug release. Therefore, gatekeeper systems using polymers like chitosan or polyethyleneimine (PEI) have been developed to regulate drug release by sealing MSN pores with stimuli-responsive nanovalves. Chitosan-functionalized MSN have demonstrated significant improvement in drug release behavior. Liu et al. demonstrated that galactosylated chitosan-functionalized MSN loaded with 5-fluorouracil (5-FU) exhibited enhanced cytotoxicity against colon cancer cells compared to amine-functionalized MSN. The study revealed that 5-FU@MSN-NH_2_ experienced rapid and uncontrolled drug release at pH 7.4, with 80% of the drug released within 0.5 h and 98% within 1.5 h. In contrast, incorporating a galactosylated chitosan (GC) shell to the 5-FU@MSN-NH_2_ resulted in significantly slower drug release rates, thereby demonstrating the effectiveness of the GC shell in providing sustained release behavior [17]. Among such gatekeepers, PEI-based systems exhibit conformational sensitivity to pH changes, enabling “on-demand” drug release triggered by acidic microenvironments, such as those found in tumors or inflamed tissues [18,19,20]. The high cationic charge density of PEI facilitates its electrostatic assembly on negatively charged MSN, tightly capping the pores and allowing swelling and uncapping around pH 5–6. Previous studies have indicated that mesoporous silica nanoparticles modified with PEI and folic acid (MSN-PEI-FA) exhibit very low release rates of curcumin in normal tissues, with release rates of 8.87% at pH 7.4, 18.52% at pH 6.8, and 54.56% at pH 5.4, thereby demonstrating its pH-sensitive and sustained release properties in tumor environments [21]. However, the potential cytotoxicity of PEI could offset the biocompatibility benefits of MSN [22]. To address this issue, imidazole-mediated modification of PEI has emerged as a promising approach to mitigate its cytotoxicity. Converting primary amine groups to imidazoles can alter toxicity profiles favorably and introduce additional pH sensitivity, making imidazole-based (IP) gatekeepers an attractive option for controlled drug delivery [23,24]. Despite this, the potential of IP nanovalves for spatiotemporally controlling celecoxib release remains underexplored. Inflammatory conditions are often associated with localized acidic environments, which are conducive to the controlled release of drugs like celecoxib from pH-sensitive delivery systems. The acidic microenvironment is a hallmark of inflamed tissues and tumors; thus, leveraging pH-responsive systems can enhance targeted drug delivery and therapeutic efficacy.

The aim of this study is to develop and evaluate dual-functionalized MSN for the targeted delivery of celecoxib. By combining amine grafting with IP gatekeepers, the objectives are to enhance drug loading capacity, achieve controlled and pH-responsive drug release, and reduce systemic toxicity. Despite the promising advancements in nanomedicine, challenges such as low drug loading capacity, uncontrolled drug release, and systemic toxicity remain significant hurdles in the development of effective drug delivery systems. Dual-functionalized MSN offer a potential solution by enhancing drug delivery performance through these synergistic modifications. This research not only addresses key limitations in current drug delivery systems but also leverages pH-responsive mechanisms to target the acidic microenvironments of inflamed tissues and tumors. By improving the therapeutic efficacy of celecoxib for both anti-inflammatory and anticancer applications, this study paves the way for future innovations in the field of targeted drug delivery systems.

## 2. Results

### 2.1. Synthesis and Characterization of Different MSN Formulations

The synthesis of MSN (MCM-41) was successfully performed using a sol–gel method with tetraethyl orthosilicate (TEOS) and cetyltrimethylammonium bromide (CTAB) as the structure-directing agent. The process was optimized to ensure uniform pore size distribution and structural integrity. Following this, MSN-NH_2_ were synthesized by APTES onto the MSN, introducing amine groups to enhance drug loading capacity. The particle size, polydispersity index (PDI), zeta potential, entrapment efficiency, and drug loading capacity of various MSN formulations were determined and are summarized in Table 1.

The particle size of the unmodified MSN (MSN-Cxb) was 218.5 ± 7.5 nm, which slightly decreased to 201.77 ± 3.74 nm upon amine functionalization (MSN-NH_2_-Cxb). This reduction in size is indicative of successful surface modification with APTES, which potentially condenses the particle surface [15]. Further modifications with PEI and imidazole groups showed varied sizes, with MSN-NH_2_-Cxb-PEI increasing to 266.90 ± 2.89 nm due to the additional polymeric coating, while MSN-NH_2_-Cxb-IP had a size of 215.60 ± 9.70 nm, suggesting a compact morphology facilitated by imidazole functionalization. The PDI values for all formulations were within the acceptable range (0.31–0.35), indicating uniform size distribution and colloidal stability (Table 1).

**Figure 1:**
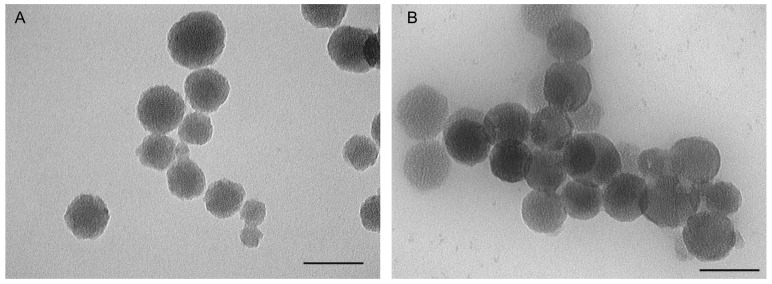
Transmission electron microscopy (TEM) results of (**A**) MSN-NH_2_-Cxb-PEI; (**B**) MSN-NH_2_-Cxb-IP. Scale bar, 100 nm.

Additionally, TEM images provided visual confirmation of the size and morphology of the nanoparticles. TEM analysis showed that MSN-NH_2_-Cxb-PEI particles exhibited an increase in size and a more irregular surface compared to MSN-NH_2_-Cxb-IP, which maintained a relatively uniform and spherical morphology. This correlates with the DLS data, confirming the structural influence of PEI and imidazole modifications (Figure 1). Moreover, the zeta potential of the unmodified MSNs was −28.79 ± 5.38 mV, reflecting the negative surface charge due to the silica network. Functionalization with APTES introduced positive amine groups, significantly shifting the zeta potential to +28.91 ± 1.81 mV for MSN-NH_2_-Cxb. This positive charge was maintained with further modifications, although slightly decreased with PEI (+26.36 ± 5.80 mV) and imidazole (+20.16 ± 3.01 mV) due to the additional functional groups altering the surface chemistry. Interestingly, amine functionalization substantially improved the entrapment efficiency and drug loading capacity of celecoxib in MSN. The entrapment efficiency increased from 6.10 ± 2.43% in MSN-Cxb to 12.89 ± 1.02% in MSN-NH_2_-Cxb, demonstrating the effectiveness of amine groups in facilitating stronger electrostatic and hydrogen bonding interactions with the drug. The loading capacity showed a similar trend, increasing from 6.18 ± 1.75% to 12.91 ± 2.02%. Further modifications with PEI and imidazole groups maintained high entrapment efficiencies (12.89 ± 1.02% and 13.17 ± 2.11%, respectively) and loading capacities (12.91 ± 2.02% and 13.51 ± 1.60%, respectively). These enhancements can be attributed to the increased surface area and functional sites provided by the dual-functionalization, which further leverage drug–nanoparticle interactions (Table 1).

The textural properties of the synthesized MSN formulations, including surface area, pore size, and pore volume, were evaluated using nitrogen adsorption–desorption isotherms. The BET surface area of MSN was found to be 204.47 m^2^·g^−1^ with a pore size of 24.32 nm and a pore volume of 2.03 cm^3^·g^−1^. Upon amine functionalization (MSN-NH_2_), the surface area decreased to 166.77 m^2^·g^−1^, the pore size reduced to 19.66 nm, and the pore volume was measured at 1.12 cm^3^·g^−1^. Further modifications with PEI showed a significant reduction in textural properties. For MSN-NH_2_-PEI, the surface area was 49.727 m^2^·g^−1^, the pore size was 10.92 nm, and the pore volume was 0.49 cm^3^·g^−1^. These results indicate successful surface modifications, as evidenced by the changes in surface area, pore size, and pore volume (Table 2).

### 2.2. TGA Results

**Figure 2:**
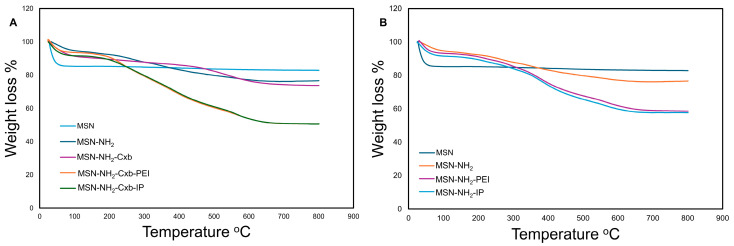
TGA curves of various MSN formulations. (**A**) TGA curves for MSN, MSN-NH_2_, MSN-NH_2_-Cxb, MSN-NH_2_-Cxb-PEI, and MSN-NH_2_-Cxb-IP. (**B**) TGA curves for MSN, MSN-NH_2_, MSN-NH_2_-PEI, and MSN-NH_2_-IP.

The thermal stability and distribution states of Cxb in the MSN formulations were assessed using TGA. The TGA curves of different formulations are shown in Figure 2. The total weight loss and Cxb-specific weight loss for each sample are summarized in Table 3. The TGA curves (Figure 2) reveal distinct decomposition patterns for each formulation. The unmodified MSN exhibited minimal weight loss up to 800 °C, indicating high thermal stability. The MSN-NH_2_-Cxb formulation showed a total weight loss of 25.68%, with 8.02% attributed to Cxb. This corresponds to a Cxb mass of 49.36 mg·g^−1^ and a Cxb mass per surface area of 0.30 mg·m^−2^. The MSN-NH_2_-Cxb-PEI formulation demonstrated a higher total weight loss of 47.90%, with 7.58% attributed to Cxb, translating to a Cxb mass of 46.69 mg·g^−1^ and a Cxb mass per surface area of 0.94 mg·m^−2^. The MSN-NH_2_-Cxb-IP formulation had the highest total weight loss of 49.80%, with 7.35% attributed to Cxb, resulting in a Cxb mass of 45.26 mg·g^−1^. These results indicate that the functionalization with PEI and imidazole groups affects the thermal stability and distribution of CXB within the MSN formulations. The higher weight loss in MSN-NH_2_-Cxb-PEI and MSN-NH_2_-Cxb-IP suggests a more substantial presence of organic content due to the functionalization, which also impacts the loading and distribution of Cxb.

### 2.3. FTIR and ^1^H-NMR Results

**Figure 3:**
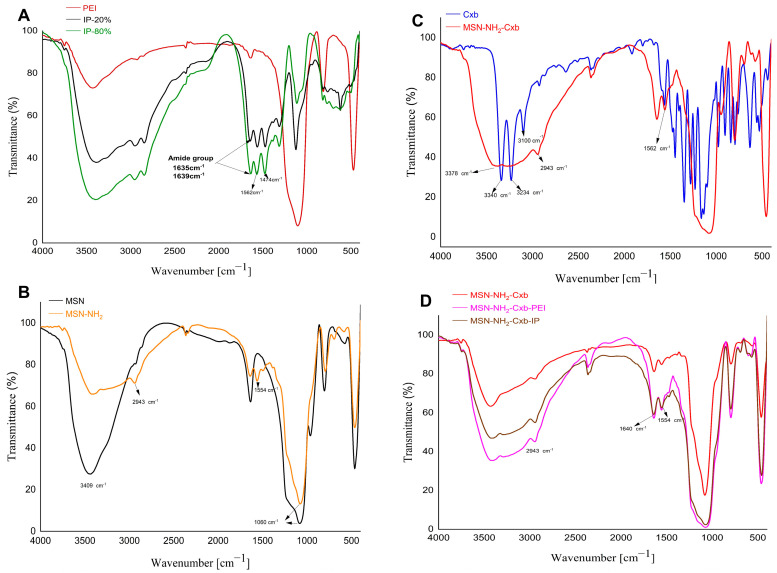
FTIR spectra of various samples. (**A**) PEI modified with 20% imidazole (IP-20%) and PEI modified with 80% imidazole (IP-80%). (**B**) Pristine MSN and amine-functionalized MSN (MSN-NH_2_). (**C**) Cxb and MSN-NH_2_ loaded with Cxb. (**D**) MSN-NH_2_-Cxb, MSN-NH_2_-Cxb-PEI, and MSN-NH_2_-Cxb-IP.

FTIR spectroscopy was employed to assess the functionalization of PEI with imidazole groups, as well as to confirm the functionalization of MSN and the conjugation of Cxb and PEI modifications. The FTIR spectra of the samples are shown in Figure 3.

To begin with, Figure 3A shows the FTIR spectrum of PEI, highlighting characteristic absorption bands. The broad peak around 3400 cm^−1^ corresponds to N-H stretching vibrations, while the peak between 1635–1639 cm^−1^ can be attributed to the deformation vibration of δ(NH), which is characteristic of the NH groups in PEI [25,26]. Additionally, the spectra of imidazole-functionalized PEI (IP-20% and IP-80%) demonstrate the successful grafting of imidazole groups onto PEI, with distinct peaks at 1562 cm^−1^ and 1474 cm^−1^. The peak at 1562 cm^−1^ is characteristic of the stretching vibrations associated with the C=C or C=N bonds within the imidazole ring, indicative of the imidazole structure’s presence [27]. Concurrently, the peak observed at 1474 cm^−1^ corresponds to the C-H bending vibrations, further substantiating the modification of PEI with imidazole groups. These intense peaks provide definitive evidence for the successful grafting of imidazole groups onto the PEI scaffold. Moving on, Figure 3B presents the FTIR spectra of pristine MSN and MSN-NH_2_. The pristine MSN spectrum exhibits characteristic peaks at 3409 cm^−1^, attributed to the O-H stretching vibrations of adsorbed water molecules, and at 1060 cm^−1^, corresponding to the Si-O-Si asymmetric stretching vibrations. Upon amine functionalization, the MSN-NH_2_ spectrum shows additional peaks at 2943 cm^−1^ and 1554 cm^−1^, indicative of C-H stretching and N-H bending vibrations, respectively [28,29]. This confirms the successful grafting of amine groups onto the MSN surface. Furthermore, Figure 3C displays the FTIR spectra of Cxb and MSN-NH_2_-Cxb. In the Cxb spectrum, characteristic peaks include 3378 cm^−1^ and 3234 cm^−1^, corresponding to N-H stretching vibrations from the sulfonamide group (NH_2_SO_2_), and 3100 cm^−1^, indicating aromatic C-H stretching vibrations. The peak at 1562 cm^−1^ corresponds to N-H bending vibrations of secondary amides. Upon loading Cxb into MSN-NH_2_, significant changes are observed. The distinct peaks at 3340 cm^−1^ and 3234 cm^−1^ disappear, replaced by a broad peak around 3378 cm^−1^, suggesting strong hydrogen bonding or electrostatic interactions between the amine groups on MSN and the N-H groups of celecoxib [30]. The peak at 2943 cm^−1^ in the MSN-NH_2_-Cxb spectrum indicates the presence of aliphatic C-H stretching vibrations, confirming the structural integrity and successful incorporation of celecoxib into the functionalized nanoparticles. In addition, Figure 3D illustrates the FTIR spectra of MSN-NH_2_-Cxb, MSN-NH_2_-Cxb-PEI, and MSN-NH_2_-Cxb-IP, providing insights into the interactions and modifications introduced by PEI and IP. The presence of a peak at 1640 cm^−1^ across all samples reflects consistent interactions involving N-H bending vibrations or C=N stretching vibrations. Notably, the peak at 1554 cm^−1^ is more pronounced in the PEI and IP samples, corresponding to N-H bending, commonly associated with the amide II band in various nitrogen-containing compounds. The amide II band typically arises from a combination of N-H bending and C-N stretching vibrations. This peak’s presence in all samples suggests nitrogen-containing functional groups in the base MSN-NH_2_-Cxb material, likely due to the NH_2_ functionalization. The peak’s increased prominence in the PEI and IP modified samples may indicate an increase in amide or amine-like functionalities following polymer modification.

**Figure 4:**
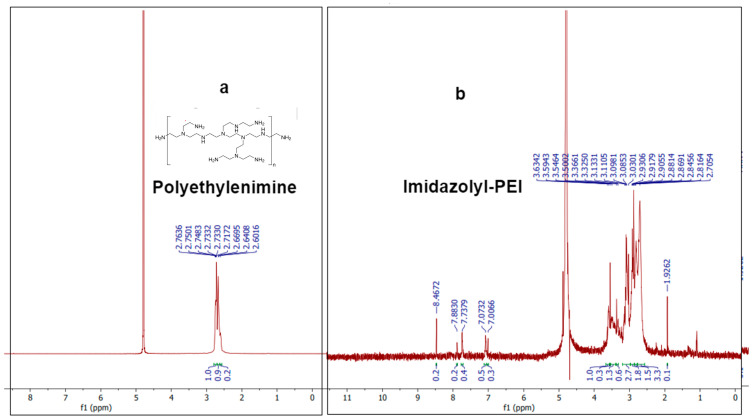
H-NMR spectra of (**a**) polyethylenimine compound and (**b**) imidazolyl-PEI (IP).

The ^1^H-NMR spectrum of PEI exhibits characteristic peaks that correspond to the chemical environment of the protons in the polymer. A prominent peak around 2.5–3.0 ppm corresponds to the methylene (-CH_2_-) protons adjacent to the amine groups in the PEI structure [31]. The ^1^H-NMR spectrum of imidazolyl-PEI (IP) shows additional peaks compared to PEI, indicating the successful conjugation of imidazole groups (Figure 4). Peaks at around 7.0–8.0 ppm are characteristic of the aromatic protons of the imidazole ring, confirming the presence of imidazole functionalities [32]. The peaks between 2.5 and 4.0 ppm can be attributed to the protons in the modified polymer backbone, indicating changes in the chemical environment due to the introduction of imidazole groups. The broad and multiple peaks suggest a more complex structure due to the functionalization process. These NMR results provide robust evidence for the successful synthesis and functionalization of PEI with imidazole groups. The characteristic peaks of the imidazole ring in the spectrum of IP, along with the shifts in the polymer backbone protons, confirm the chemical modifications achieved in the synthesis process.

### 2.4. In Vitro Release Testing

**Figure 5:**
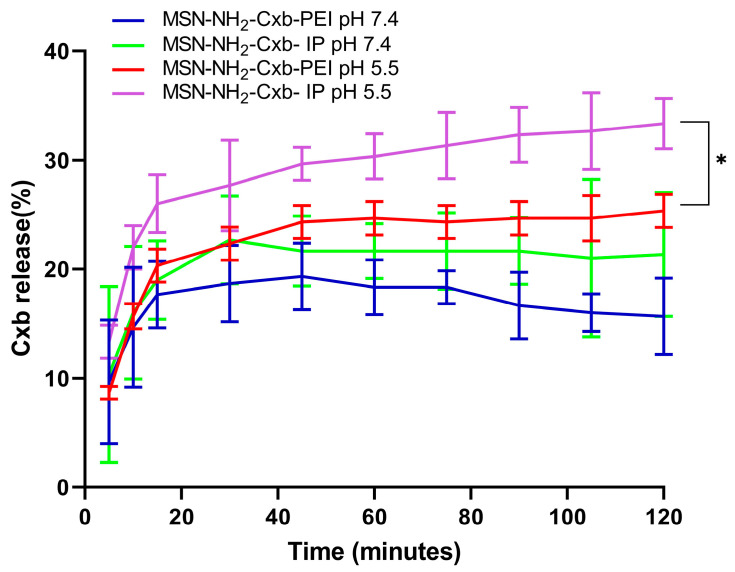
Release profile of Cxb from MSN-NH_2_-Cxb-PEI/IP in pH 5.5 and pH 7.4 media. Data are presented as mean ± SD (n = 3). Statistical significance was determined using a paired *t*-test: * *p* < 0.05, for MSN-NH_2_-Cxb-IP at pH 5.5 compared to MSN-NH_2_-Cxb-PEI at pH 5.5.

The release profile of celecoxib from MSN functionalized with PEI and IP at different pH levels (7.4 and 5.5) demonstrates significant differences in drug release behavior, as shown in Figure 5. The formulations compared include MSN-NH_2_-Cxb-PEI at pH 7.4 (blue line), MSN-NH_2_-Cxb-IP at pH 7.4 (green line), MSN-NH_2_-Cxb-PEI at pH 5.5 (red line), and MSN-NH_2_-Cxb-IP at pH 5.5 (purple line). The data indicate that the release of celecoxib is significantly influenced by both the pH of the environment and the type of functionalization of the nanoparticles. At pH 7.4, the release profile of MSN-NH_2_-Cxb-PEI shows a controlled and gradual release of celecoxib, reaching about 15.7 ± 3.51% after 120 min. This steady release indicates stability and slower drug liberation under physiological conditions. The imidazolyl modification shows a slightly higher release, peaking at around 21.3 ± 5.7% after 120 min. This slight enhancement is likely due to better interaction with the aqueous medium and increased hydrophilicity. In the acidic environment (pH 5.5), simulating both the endosomal and inflammatory environment of cells, the release of celecoxib from PEI-functionalized MSN is more pronounced, reaching approximately 28.8 ± 1.52% after 120 min. The increased release at acidic pH can be attributed to the protonation of the amine groups in PEI, which enhances solubility and drug release. Interestingly, MSN-NH_2_-Cxb-IP show the highest release, with 33.3 ± 2.3% of celecoxib released after 120 min. The imidazole groups’ higher proton sponge capacity leads to a faster and greater release in acidic conditions, highlighting the efficacy of imidazolyl modification in promoting drug release under endosomal and inflammatory conditions.

When comparing the release profiles at pH 5.5 using a paired *t*-test, there is a statistically significant increase in the release of celecoxib from MSN-NH_2_-Cxb-IP compared to MSN-NH_2_-Cxb-PEI (*p* < 0.044). Specifically, after 120 min, the release from MSN-NH_2_-Cxb-PI at pH 5.5 is significantly higher (33.3 ± 2.3%) compared to MSN-NH_2_-Cxb-PEI at pH 5.5 (28.8 ± 1.52%). This significant difference underscores the effectiveness of the imidazolyl modification in enhancing drug release in acidic environments. The enhanced release at pH 5.5 suggests that the imidazolyl-PEI (IP) gatekeepers are more responsive to pH changes, significantly increasing the rate and amount of drug released. The imidazole ring’s protonation in acidic conditions increases the drug release, making the MSN-NH_2_-Cxb-IP formulation more effective for targeted drug delivery in acidic environments such as endosomal, inflammatory, or cancer conditions.

### 2.5. Cell Viabilty

**Figure 6:**
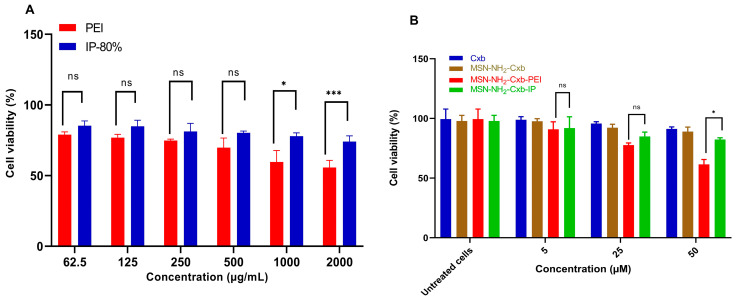
(**A**) Cell viability of RAW 264.7 cells treated with various concentrations of PEI and IP 80% (62.5, 125, 250, 500, 1000, and 2000 µg/mL) after 24 h. (**B**) Cell viability of RAW 264.7 cells treated with Cxb and mesoporous silica nanoparticles loaded with Cxb (MSN-NH_2_-Cxb, MSN-NH_2_-Cxb-PEI, and MSN-NH_2_-Cxb-IP) at concentrations of 5, 25, and 50 µM after 24 h. Data are presented as mean ± SD (n = 3). Statistical significance was determined using a paired *t*-test. For (**A**), IP-80% compared to PEI: * *p* < 0.05, *** *p* < 0.001, ns: not significant. For (**B**), MSN-NH_2_-Cxb-IP compared to MSN-NH_2_-Cxb-PEI: * *p* < 0.05, ns: not significant.

The cell viability and cytotoxicity of PEI and IP at 80% modification were evaluated across a concentration range from 62.5 to 2000 µg/mL using RAW 264.7 cells. The results demonstrate that PEI imidazole at 80% modification exhibits reduced cytotoxicity compared to unmodified PEI at higher concentrations (Figure 6A).

At a concentration of 1000 µg/mL, IP 80% showed significantly higher cell viability compared to PEI, with a *p*-value of less than 0.033. At the highest concentration tested, 2000 µg/mL, IP 80% also demonstrated significantly higher cell viability compared to PEI, with a *p*-value of less than 0.001. These results indicate that the imidazolyl modification effectively attenuates the cytotoxicity of PEI at higher concentrations. While there was an observed increase in cell viability for IP 80% at lower concentrations (62.5 to 500 µg/mL), these differences were not statistically significant (*p* ≥ 0.05). These results indicate that the imidazolyl modification effectively attenuates the cytotoxicity of PEI at higher concentrations. Further evaluation of Cxb and the mesoporous silica nanoparticles loaded with celecoxib (MSN-NH_2_-Cxb, MSN-NH_2_-Cxb-PEI, and MSN-NH_2_-Cxb-IP) supports these findings (Figure 6B). At concentrations of 5 and 25 µM, MSN-NH_2_-Cxb-IP showed non-significant differences in cell viability with *p*-values of 0.116 and 0.192, respectively. However, at a concentration of 50 µM, MSN-NH_2_-Cxb-IP exhibited a significant increase in cell viability with a *p*-value of 0.014. This pattern aligns with the results observed for PEI and IP, where the cytotoxicity-reducing effect is more pronounced at higher concentrations. These findings reveal that the imidazolyl modification of PEI not only enhances drug release properties, as shown in previous sections, but also reduces cytotoxicity at higher concentrations. Similarly, the loading of Cxb into MSN-NH_2_, and further modifications with PEI and IP, demonstrates a reduction in cytotoxicity, particularly at higher concentrations. This dual benefit makes imidazolyl-PEI and MSN-NH_2_-Cxb loaded nanoparticles promising candidates for drug delivery applications, offering improved biocompatibility and efficacy.

### 2.6. Evaluation of Anti-Inflammatory Effects Using NO Assay

**Figure 7:**
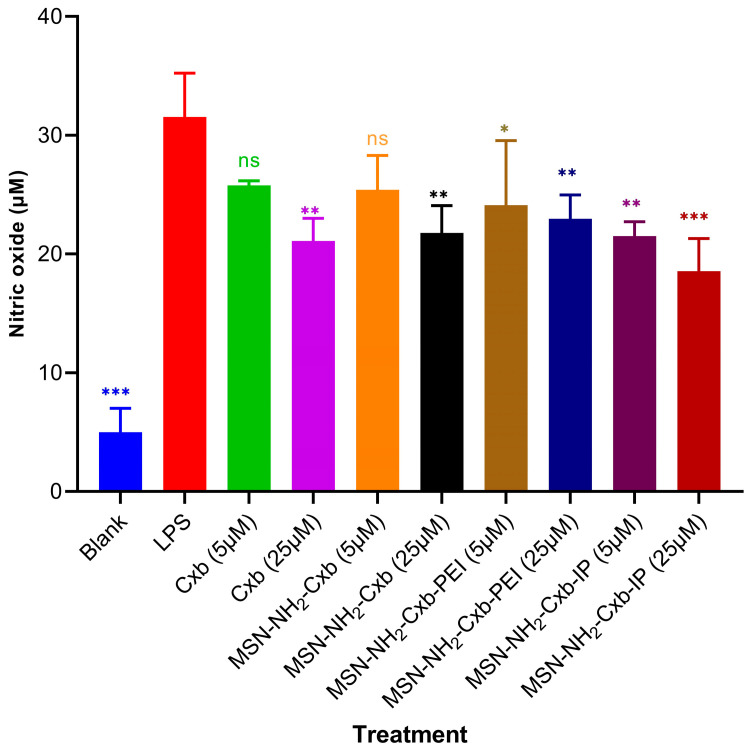
NO production in RAW 264.7 cells treated with various formulations. Cells were stimulated with LPS (1 µg/mL) and treated with free Cxb at 5 and 25 µM, and MSN formulations loaded with Cxb (MSN-NH_2_-Cxb, MSN-NH_2_-Cxb-PEI, MSN-NH_2_-Cxb-IP) at 5 and 25 µM. Data are presented as mean ± SD (n = 3). Statistical significance was determined using one-way ANOVA followed by Dunnett’s test comparing samples with LPS treatment: * *p* < 0.05, ** *p* < 0.01, *** *p* < 0.001; non-significant (ns) for *p* ≥ 0.05.

The anti-inflammatory effects of the MSN formulations were assessed by measuring NO production in RAW 264.7 cells, as shown in Figure 7. Exposure to LPS (1 µg/mL) induced the highest NO production, confirming the successful induction of inflammation. In contrast, the blank control showed significantly lower NO production compared to cells treated with LPS alone (*** *p* < 0.001).

Treatment with free Cxb at 5 µM did not yield a statistically significant reduction in NO levels relative to the LPS-treated group (ns, *p* > 0.05). However, at a higher concentration of 25 µM, Cxb significantly attenuated NO production (** *p* < 0.01), demonstrating a dose-dependent anti-inflammatory effect. Similarly, treatment with MSN-NH_2_-Cxb at 5 µM did not significantly affect NO levels (ns, *p* > 0.05), whereas at 25 µM, a significant reduction in NO production was observed (** *p* < 0.01). This underscores the efficacy of the nanocarrier at higher drug loads in mitigating inflammation. Furthermore, MSN-NH_2_-Cxb-PEI demonstrated significant anti-inflammatory activity, as evidenced by the reduction in NO levels at both 5 µM (* *p* < 0.05) and 25 µM (** *p* < 0.01), compared to LPS alone. This suggests that PEI modification enhances the therapeutic potential of the formulation. The most pronounced anti-inflammatory effect was exhibited by MSN-NH_2_-Cxb-IP, which significantly reduced NO production at both 5 µM (** *p* < 0.01) and 25 µM (*** *p* < 0.001). The imidazolyl modification appears to potentiate the anti-inflammatory properties of the MSN formulations, making MSN-NH_2_-Cxb-IP the most effective among the tested variants.

## 3. Discussion

The dual-functionalization of MSN with amine grafting and IP gatekeepers presents significant advancements in the targeted delivery of hydrophobic drugs such as celecoxib. The enhanced loading capacity and controlled release observed in this study underscore the efficacy of this approach. The successful synthesis of MSN using the sol–gel method ensured uniform pore size distribution and structural integrity, crucial for effective drug delivery systems. Amine functionalization with APTES was indicated by the presence of amine groups and a slight reduction in particle size, from 218.5 ± 7.5 nm to 201.77 ± 3.74 nm, observed through characterization techniques. This reduction in size may result from the condensation of the particle surface during functionalization, potentially due to a more compact arrangement of the silica network [33,34].

Amine functionalization significantly increased the loading capacity of celecoxib in MSN. The grafting of APTES onto the MSN introduced a high density of amine groups, which facilitated stronger electrostatic and hydrogen bonding interactions with the hydrophobic drug molecules. This modification expanded the pore volume and diameter, providing more space for drug molecules and enhancing their interaction with the amine-functionalized surface [35]. Similar enhancements in drug loading have been reported in previous studies. For example, Manzano et al. achieved a 10% increase in ibuprofen loading with amine-grafted MSN, and Ahmadi et al. reported an 11% increase in ibuprofen loading capacity with aminopropyl-functionalized MSN [36,37]. These findings align with our observations of a 2.1-fold increase in celecoxib loading capacity, attributed to the increased surface area and pore volume, and the presence of amine groups that facilitate better interaction with the drug molecules.

Previous studies have reported varying entrapment efficiencies of celecoxib in silica nanoparticles, with values ranging from 5.7 to 12.4%. Notably, Kim et al. demonstrated that the highest entrapment capacity of 12.4% was achieved using the incipient wetness impregnation method to load celecoxib onto MCM-41 silica nanoparticles [38]. In contrast, other studies have reported lower entrapment efficiencies, such as 5.7% and 11.9%, attributed to the use of methanolic and ethanolic preparations for loading celecoxib into SBA-15-A silica nanoparticles [14]. These differences may be due to various factors, including the type of silica nanoparticles used, the drug loading method, and the conditions under which the process occurs. The relatively low entrapment efficiency observed in many studies, including ours, can be explained by the large molecular size of celecoxib, which may limit its diffusion into the nanopores. Additionally, the hydrophobic nature of celecoxib can lead to aggregation and poor dispersibility in the aqueous medium used during the loading process. Despite these challenges, our study demonstrated an entrapment efficiency of approximately 12.89 to 13.17%, which is comparable to or better than the values reported in previous studies.

Further surface modification using pH-sensitive polymers like PEI could enhance the targeting capabilities of silica nanoparticles through acidic environments such as endosomes, inflammatory tissues, and cancerous cells. However, it is well known that PEI suffers from toxicity concerns. To address this, we aimed to simultaneously boost pH sensitivity to increase drug release in acidic environments and attenuate the toxicity of PEI through IP. This dual approach, often described as hitting two birds with one stone, leverages the proton sponge effect of PEI for enhanced release in acidic conditions while reducing PEI toxicity. The imidazolyl modification of PEI introduced a proton sponge effect, which significantly enhanced drug release in acidic environments such as endosomes, inflammatory tissues, and tumors. The release profiles demonstrated that MSN-NH_2_-Cxb-IP showed a significantly higher release at pH 5.5 compared to MSN-NH_2_-Cxb-PEI, confirming the effectiveness of the imidazolyl modification. The statistical analysis further supported the significance of these findings (*p* < 0.044). This enhanced release can be attributed to the higher proton sponge capacity of imidazole groups, which facilitates the swelling and uncapping of the nanoparticle pores in acidic conditions, leading to faster and greater drug release. Our cytotoxicity results revealed a significantly decreased cytotoxic effect on RAW 264.7 cells when treated with IP compared to PEI after 24 h. This decrease in cytotoxicity was statistically significant at high concentrations (1000 and 2000 µg/mL), aligning with previous studies that demonstrated a reduction in PEI toxicity at high concentrations through aromatic amino acid modifications [39]. The reduction in cytotoxicity can be potentially explained by several mechanisms. PEI is highly cationic, which can cause membrane disruption and cytotoxicity. The imidazolyl modification reduces the overall positive charge density, thereby minimizing the electrostatic interactions with cell membranes, leading to lower cytotoxicity. Additionally, the imidazole rings are known to be more biocompatible than primary amines, which mitigates the toxic effects associated with primary amines in PEI [40].

Our anti-inflammatory assessment of free Cxb and various MSN formulations was conducted using the NO production assay. We selected two doses, 5 and 25 µM, for Cxb in all formulations, as these concentrations demonstrated high cell viability in our preliminary cell viability tests. Our results revealed that both Cxb and MSN-NH_2_-Cxb significantly decreased NO production in treated cells compared to cells treated with LPS alone, specifically at the higher concentration of 25 µM. This indicates the anti-inflammatory efficacy of these formulations at higher doses. In contrast, the modification with PEI significantly decreased NO production at the low dose of 5 µM. Interestingly, the modification with IP exhibited the most pronounced effect in decreasing NO production, revealing the highest anti-inflammatory activity among all treatments. This enhanced effect is potentially attributable to the properties of PEI, which include high cellular uptake and effective endosomal escape via the proton sponge effect [41]. Additionally, the imidazole groups play a crucial role in enhancing these properties. Previous studies have shown that imidazole groups, due to their pH-sensitive properties, can absorb and neutralize protons, preventing endosomal acidification. This buffering action causes the endosomal membrane to swell, maximizing the proton sponge effect, which leads to successful endosomal escape and the release of imidazole-bearing particles into the cytosol [42]. Consequently, the modification with IP may synergistically enhance endosomal escape and cellular uptake, thereby contributing to the superior anti-inflammatory efficacy observed. Furthermore, the acidic environment of inflammation can enhance the release of the therapeutic agent from the MSN formulations. The pH-sensitive imidazole groups respond to the acidic conditions, facilitating higher drug release specifically at the site of inflammation. This targeted release mechanism, combined with improved cellular uptake and endosomal escape, likely explains the superior anti-inflammatory effects of the MSN-NH_2_-Cxb-IP formulation. These findings highlight the potential of functionalized MSN formulations for targeted anti-inflammatory therapy. The ability of MSN-NH_2_-Cxb-IP to significantly reduce NO production at both low and high doses underscores its promise as a potent anti-inflammatory agent. Imidazolyl PEI modification not only enhances therapeutic efficacy but also reduces toxicity, offering a promising strategy for improving therapeutic outcomes.

This study provides a robust framework for enhancing the loading capacity and controlled release of hydrophobic drugs, addressing key challenges in drug delivery such as low loading capacity, uncontrolled release, and systemic toxicity. The innovative approach of dual-functionalizing MSN with amine grafting and imidazolyl-PEI gatekeepers has demonstrated significant improvements in drug delivery efficiency and biocompatibility, offering a promising strategy for the treatment of inflammatory and cancerous conditions. Moreover, the dual-functionalization significantly enhances the anti-inflammatory therapeutic effect by ensuring efficient delivery and release of the drug at the site of inflammation. Future research should focus on optimizing the balance between drug loading capacity, release kinetics, and cytotoxicity. Exploring other functional groups and polymers that can be grafted onto MSN could further enhance their drug delivery capabilities. Additionally, in vivo studies are necessary to evaluate the therapeutic efficacy and safety of these nanoparticles in clinical settings. The development of multifunctional MSN with the ability to target specific cells and tissues, respond to multiple stimuli, and deliver a combination of therapeutic agents could pave the way for more effective and personalized treatments for various diseases. The advancements presented in this study set the stage for future innovations in the field of targeted drug delivery systems.

## 4. Materials and Methods

### 4.1. Materials

The following chemicals were obtained from Sigma Aldrich (St. Louis, MO, USA): CTAB, TEOS, ammonia, ethanol, distilled water, APTES, branched PEI (average MW 750 kDa), 4-imidazoleacetic acid hydrochloride, l-ethyl-3-(3-dimethylaminopropyl) carbodiimide (EDAC), Na_2_HPO4, NaH_2_PO_4_, glacial acetic acid, and sodium lauryl sulfate. Celecoxib was purchased from PT. Novell Pharmaceutical Laboratories (Bogor, Indonesia), and sodium acetate from Merck (Rahway, NJ, USA). Additional materials used in this study include 3-(4,5-dimethylthiazol-2-yl)-2,5-diphenyltetrazolium bromide (MTT) purchased from Thermo Fisher Scientific (Waltham, MA, USA) for cytotoxicity assays, and high retention dialysis tubing with a molecular weight cut-off of 12.4 kDa purchased from Sigma-Aldrich (St. Louis, MO, USA) for dialysis processes.

### 4.2. Synthesis of Mesoporous Silica Nanoparticles (MSN)

The synthesis of MSN was performed by the sol–gel method as described by Ashour et al. with minor modifications [43]. Briefly, 200 mg of CTAB was dissolved in 70 mL of distilled water and 20 mL of ethanol in a 100 mL Erlenmeyer flask. The solution was stirred, and 270 µL of ammonia was added. The mixture was stirred at 200 rpm and 40 °C in a water bath shaker. After 30 min, 1 mL of TEOS was added in 40 increments of 25 µL each [44]. Stirring continued for 24 h. The resulting MSN suspension was centrifuged at 5000× *g* rpm for 15 min. The supernatant was discarded, and the precipitate was washed with ethanol and dried at 40 °C for 24 h. The dried MSNs were then calcined at 550 °C for 6 h.

### 4.3. Functionalization and Drug Loading in MSN

**Figure 8:**
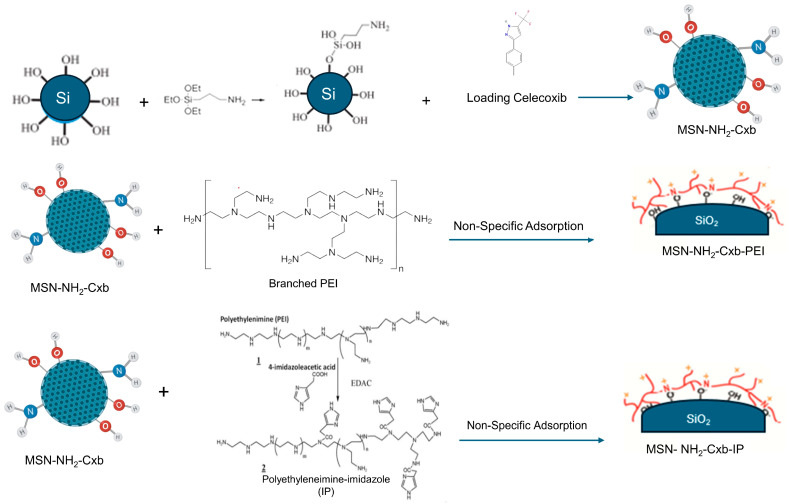
Schematic representation of the synthesis and functionalization of MSN with amine groups, loading with celecoxib (Cxb), and subsequent modifications with branched PEI and IP.

MSNs were synthesized and functionalized with amine groups using APTES to form MSN-NH_2_. The functionalized MSNs were then loaded with Cxb to create MSN-NH_2_-Cxb. Post-synthetic modifications were performed to further enhance drug delivery properties: MSN-NH_2_-Cxb was modified with branched PEI and polyethylenimine-imidazole (IP) via non-specific adsorption, resulting in MSN-NH2-Cxb-PEI and MSN-NH_2_-Cxb-IP, respectively. The functionalization and modification steps are illustrated schematically in Figure 8.

Functionalization and drug loading followed established protocols [44]. In brief, 1 mL of APTES was added to 19 mL of ethanol in a vial. A quantity of 100 mg of MSN was dispersed in the mixture, which was stirred at 200 rpm and 25 °C in a water bath shaker for 24 h. The mixture was centrifuged at 5000× *g* rpm for 15 min. The supernatant was discarded, and the precipitate was washed with ethanol and dried in an oven at 60 °C for several hours to obtain MSN-NH_2_ (yield, 60%). For drug loading, 100 mg of celecoxib was dissolved in 5 mL of ethanol and sonicated for 30 min. A quantity of 100 mg of MSN-NH_2_ was added to the celecoxib solution and stirred with a magnetic stirrer at 400 rpm at room temperature for 24 h. The mixture was centrifuged at 5000× *g* rpm for 15 min. The supernatant was collected for analysis, and the precipitate was resuspended in distilled water. The MSN-NH_2_-Cxb (yield, 75%) solid was dried in an oven at 40 °C for 24 h.

### 4.4. Synthesis and Conjugation of IP to MSN-NH_2_-Cxb

IP was synthesized following the protocol adapted from previously described methods for conjugating functional groups to polyethyleneimines [45]. Briefly, 56.4 mg of 4-imidazoleacetic acid hydrochloride, sufficient to achieve 80% substitution of the primary amine groups in PEI, was dissolved in 1 mL of water. To adjust the pH, 7.33 mg of sodium bicarbonate was added and stirred for 5 min. This solution was then slowly added dropwise to a vigorously stirred 1 mg/mL solution of PEI at room temperature. After 1 h of stirring, 32 mg of EDAC was introduced to facilitate the coupling reaction. The mixture was stirred overnight to ensure complete conjugation. The reaction mixture was then concentrated using a rotary evaporator to reduce the volume to one-third of its original volume, and subsequently transferred into a dialysis bag. Dialysis against water was performed for 48 h with periodic water changes to remove unreacted components, followed by lyophilization to obtain IP. To conjugate the pH-responsive gatekeeper to MSN-NH_2_-Cxb, 60 mg of MSN-NH_2_-Cxb was dispersed in 5 mL of distilled water and sonicated in a sonication bath for 30 min to ensure uniform suspension. Subsequently, 5 mL of a 0.2% PEI solution was added to the suspension and stirred at 400 rpm at room temperature for 24 h. The mixture was then centrifuged at 5000× *g* rpm for 15 min, and the precipitate was washed thoroughly with distilled water to remove any unbound polymer. The nanoparticles were dried in an oven at 40 °C for 12–24 h. The yield of MSN-NH_2_-Cxb-PEI was approximately 70%. The same protocol was followed to conjugate IP to the MSN-NH_2_-Cxb, ensuring the pH-responsive gatekeeper was effectively attached. The yield of MSN-NH_2_-Cxb-IP was approximately 65%.

### 4.5. Characterization of Nanoparticles

The particle size, PDI, and zeta potential of the synthesized MSN suspended in distilled water at neutral pH 7.2 were measured using a particle size analyzer (Beckman Coulter, West Sacramento, CA, USA) and a zeta potential analyzer (Malvern Instruments, Malvern, UK), respectively. The morphology and size of the synthesized nanoparticles were analyzed using TEM. Samples were prepared by placing a drop of the nanoparticle suspension onto a copper grid, followed by drying under ambient conditions. TEM images were captured to assess particle size, shape, and surface morphology. The surface area and pore volume of the synthesized MSN formulations were determined using nitrogen adsorption–desorption isotherms at 77 K, measured with a Micromeritics ASAP 2020 system. The samples were degassed under vacuum at 120 °C for 12 h prior to analysis. The Brunauer–Emmett–Teller (BET) method was used to calculate the specific surface area, while the total pore volume was estimated from the amount of nitrogen adsorbed at a relative pressure (P/P0) of 0.99.

### 4.6. Thermogravimetric Analysis (TGA)

Thermal analysis was conducted using a STA 7300 simultaneous thermal analyzer (Hitachi High-Tech Science Corporation, Tokyo, Japan). Approximately 5–10 mg of each MSN sample was placed into platinum crucibles and subjected to heating from 25 °C to 800 °C at a rate of 10 °C/min under a 30 mL/min nitrogen atmosphere. An empty aluminum oxide (Al_2_O_3_) crucible served as the reference [46]. The weight loss was recorded as a function of temperature to analyze the decomposition profiles and quantify the Cxb content.

### 4.7. FTIR and ^1^H-NMR Characterization

FTIR and ^1^H-NMR analyses were conducted to confirm the successful synthesis of IP. Additionally, the FTIR spectra of pristine MSN, MSN-NH_2_, Cxb, MSN-NH_2_-Cxb, and MSN-NH_2_-Cxb further modified with PEI (MSN-NH_2_-Cxb-PEI) and imidazole-modified PEI (MSN-NH_2_-Cxb-IP) were analyzed and compared. FTIR spectra were recorded using a Jasco FT/IR-4200 type A spectrometer (Jasco Corporation, Tokyo, Japan). Samples were prepared by mixing the synthesized nanoparticles with potassium bromide (KBr) and compressing them into pellets. The spectra were obtained in the range of 4000–400 cm^−1^. For H-NMR analysis, the samples were dissolved in deuterated dimethyl sulfoxide (DMSO-d6) and analyzed using a Bruker NMR spectrometer (Bruker Corporation, Billerica, MA, USA). Chemical shifts were recorded in parts per million (ppm) relative to tetramethylsilane (TMS) as an internal standard. The presence of characteristic peaks corresponding to the imidazole and polyethyleneimine groups was used to confirm the structure of the synthesized IP.

### 4.8. Determination of Entrapment Efficiency and Loading Capacity

The supernatant from the celecoxib loading process was diluted 2000-fold and analyzed using a UV–visible spectrophotometer (PG instruments, Lutterworth, UK) at 253 nm to determine the amount of unentrapped celecoxib, following the method of Alaaeldin et al. [47]. The following equations were used to calculate the entrapment efficiency and loading capacity:

$$\mathrm{Entrapment}\;\mathrm{Efficiency}\;(\%)=\frac{\mathrm{Initial}\;\mathrm{amount}\;\mathrm{of}\;\mathrm{Cxb}-\;\mathrm{amount}\;\mathrm{of}\;\mathrm{Cxb}\;\mathrm{unentrapped}\;}{\mathrm{Intial}\;\mathrm{amount}\;\mathrm{of}\;\mathrm{Cxb}\;}\times 100\%$$

$$\mathrm{Loading}\;\mathrm{Capacity}\;(\%)\;=\frac{\mathrm{Initial}\;\mathrm{amount}\;\mathrm{of}\;\mathrm{Cxb}-\;\mathrm{amount}\;\mathrm{of}\;\mathrm{Cxb}\;\mathrm{unentrapped}}{\mathrm{Amount}\;\mathrm{of}\;\mathrm{MSN}\;\mathrm{added}}\times 100\%$$

### 4.9. In Vitro Release Testing

In vitro release testing was conducted following the methods described by Kim et al. [48]. Briefly, 100 mg samples of MSN-NH_2_ functionalized with PEI and IP gatekeepers, each containing 1 mg of celecoxib, were placed in vials containing 30 mL of either pH 7.4 phosphate buffer or pH 5.5 acetate buffer. The samples were stirred at 100 rpm and 37 °C in a water bath shaker. At specific time intervals (5, 10, 15, 30, 45, 60, 75, 90, 105, and 120 min), 5 mL aliquots were withdrawn and replaced with fresh buffer to maintain sink conditions. The withdrawn samples were filtered using a 0.45 µm membrane filter to remove any particulate matter. The filtrate’s absorbance was measured at 253 nm using a T92+ UV–visible spectrophotometer (PG Instruments, Lutterworth, UK) to determine the concentration of released celecoxib. The use of pH 7.4 buffer simulates the extracellular or typical cell environment, while the pH 5.5 buffer mimics the acidic conditions of the endosomal environment in inflammatory cells [49]. To prevent aggregation and maintain sink conditions, sodium lauryl sulfate (SLS) was added to the testing media as a surfactant. The total testing duration of two hours was chosen based on the required time for endosomal escape, as demonstrated in earlier studies [50,51].

### 4.10. Cell Culture

RAW 264.7 cells were cultured in Dulbecco’s Modified Eagle Medium (DMEM) supplemented with 10% fetal bovine serum (FBS) and 1% penicillin-streptomycin. The cells were incubated at 37 °C with 5% CO_2_ until confluent. The cells were then washed with phosphate-buffered saline (PBS) and harvested using trypsin-EDTA.

### 4.11. Cell Viability Assay

The cell viability and cytotoxicity tests were performed based on the method described by Loan et al. with modifications [52]. In brief, RAW 264.7 cells (1 × 10^4^ cells per well) were seeded into a 96-well plate with 100 µL of culture medium per well and incubated at 37 °C with 5% CO_2_ for 24 h. The medium was then removed, and six dilutions of PEI and IP samples in culture medium (100 µL per well) were added to the wells. Control wells received only culture medium. Additionally, Cxb and mesoporous silica nanoparticles loaded with celecoxib (MSN-NH_2_-Cxb, MSN-NH_2_-Cxb-PEI, and MSN-NH_2_-Cxb-IP) were tested at concentrations of 5, 25, and 50 µM. After 24 h of incubation, the medium was removed, and the cells were washed with PBS. MTT reagent (0.5 mg/mL) in DMEM was added to the wells (100 µL per well), and the cells were incubated for 4 h at 37 °C. After incubation, the MTT solution was removed, and 100 µL of dimethyl sulfoxide (DMSO) was added to each well to dissolve the formazan crystals. The absorbance was measured at 570 nm using a microplate reader. The experiments were performed in triplicate and the results are expressed as mean ± SD.

### 4.12. Nitric Oxide (NO) Assay

The anti-inflammatory effects of the MSN formulations were evaluated by measuring nitric oxide (NO) production in RAW 264.7 cells using the Griess reagent (Sigma Aldrich, St. Louis, MO, USA). RAW 264.7 cells (1 × 10^5^ cells per well) were seeded into a 96-well plate and incubated at 37 °C with 5% CO_2_ for 24 h. The cells were then treated with lipopolysaccharide (LPS) at a concentration of 1 µg/mL to induce inflammation. Various treatments were applied, including free Cxb at 5 and 25 µM, and MSN formulations loaded with celecoxib (MSN-NH_2_-Cxb, MSN-NH_2_-Cxb-PEI, and MSN-NH_2_-Cxb-IP) at 5 and 25 µM. Blank wells, containing cells but no treatment, were included as a control. After 24 h of incubation with the treatments, the culture supernatants were collected, and NO production was measured using the Griess reagent. Briefly, 100 µL of supernatant was mixed with 100 µL of Griess reagent (1% sulfanilamide and 0.1% N-(1-naphthyl) ethylenediamine dihydrochloride in 2.5% phosphoric acid) in a 96-well plate. The mixture was incubated at room temperature for 10 min, and the absorbance was measured at 540 nm using a microplate reader. The experiments were performed in triplicate, and the results are expressed as mean ± SD

### 4.13. Statistical Analysis

Statistical analysis was performed to evaluate the significance of the differences observed in the release profiles and cell viability assays. Data are presented as mean ± standard deviation (SD) from three independent experiments (n = 3). The statistical significance of differences between groups was determined using paired *t*-tests and analyzed using GraphPad Prism 8 software.

## 5. Conclusions

The successful synthesis and dual-functionalization of MSN with amine grafting and imidazolyl PEI gatekeepers demonstrate significant advancements in targeted drug delivery. The imidazolyl modification effectively reduces the inherent cytotoxicity of unmodified PEI, enhancing biocompatibility and reducing dose-dependent toxicity, and enhancing the anti-inflammatory therapeutic effect. The amino-functionalized MSN exhibited favorable characteristics, including increased drug loading capacity and pH-responsive behavior. In vitro release studies confirmed the efficacy of the imidazolyl PEI gatekeeper in achieving controlled, pH-triggered drug release. This innovative system addresses key challenges in NSAID delivery, reducing side effects and improving therapeutic outcomes for chronic inflammatory conditions. Future research should focus on optimizing these systems and conducting in vivo studies to evaluate therapeutic efficacy and safety.

## Figures and Tables

**Table 1:** Characteristics of nanoparticles.

Characteristics	Type of Nanoparticles			
	MSN-Cxb	MSN-NH_2_-Cxb	MSN-NH_2_-Cxb-PEI	MSN-NH_2_-Cxb-IP
Particle size (nm)	218.5 ± 7.50	201.77 ± 3.74	266.90 ± 2.89	215.60 ± 9.70
Polydispersity index	0.34 ± 0.05	0.31 ± 0.01	0.32 ± 0.01	0.35 ± 0.08
Zeta potential (mV)	−28.79 ± 5.38	28.91 ± 1.81	26.36 ± 5.79	20.16 ± 3.01
Entrapment efficiency (%)	6.10 ± 2.43	12.89 ± 1.02	12.89 ± 1.02	13.17 ± 2.11
Loading capacity (%)	6.18 ± 1.75	12.91 ± 2.02	12.91 ± 2.02	13.51 ± 1.60

*n* = 3, Cxb: celecoxib.

**Table 2:** Surface area, pore size, and pore volume of various MSN formulations.

Sample	Surface Area [m^2^·g^−1^]	Pore Size (nm)	Pore Volume (cm^3^·g^−1^)
MSN	204.47	24.32	2.03
MSN-NH_2_	166.77	19.66	1.12
MSN-NH_2_-PEI	49.727	10.92	0.49

**Table 3:** Distribution and amount of Cxb in MSN formulations from TGA results.

Sample	Total Weight Loss wt.%	Cxb Weight Loss wt.%	Cxb Mass [mg·g^−1^]	Cxb Mass per Surface [mg·m^−2^]
MSN-NH_2_-Cxb	25.68	8.02	49.36	0.30
MSN-NH_2_-Cxb-PEI	47.90	7.58	46.69	0.94
MSN-NH_2_-Cxb-IP	49.80	7.35	45.26	-

## Data Availability

Data are contained within the article.
